# A role for artificial intelligence applications inside and outside of the operating theatre: a review of contemporary use associated with total knee arthroplasty

**DOI:** 10.1186/s42836-023-00189-0

**Published:** 2023-07-04

**Authors:** Andrew P. Kurmis

**Affiliations:** 1 Discipline of Medical Specialties, University of Adelaide, Adelaide, SA 5005 Australia; 2 Department of Orthopaedic Surgery, Lyell McEwin Hospital, Haydown Road, Elizabeth Vale, SA 5112 Australia; 3 College of Medicine & Public Health, Flinders University, Bedford Park, SA 5042 Australia

**Keywords:** Artificial intelligence (AI), Deep learning, Knee arthroplasty, Knee replacement, Machine learning

## Abstract

**Background:**

Artificial intelligence (AI) has become involved in many aspects of everyday life, from voice-activated virtual assistants built into smartphones to global online search engines. Similarly, many areas of modern medicine have found ways to incorporate such technologies into mainstream practice. Despite the enthusiasm, robust evidence to support the utility of AI in contemporary total knee arthroplasty (TKA) remains limited. The purpose of this review was to provide an up-to-date summary of the use of AI in TKA and to explore its current and future value.

**Methods:**

Initially, a structured systematic review of the literature was carried out, following PRISMA search principles, with the aim of summarising the understanding of the field and identifying clinical and knowledge gaps.

**Results:**

A limited body of published work exists in this area. Much of the available literature is of poor methodological quality and many published studies could be best described as “demonstration of concepts” rather than “proof of concepts”. There exists almost no independent validation of reported findings away from designer/host sites, and the extrapolation of key results to general orthopaedic sites is limited.

**Conclusion:**

While AI has certainly shown value in a small number of specific TKA-associated applications, the majority to date have focused on risk, cost and outcome prediction, rather than surgical care, per se. Extensive future work is needed to demonstrate external validity and reliability in non-designer settings. Well-performed studies are warranted to ensure that the scientific evidence base supporting the use of AI in knee arthroplasty matches the global hype.

## Background

Artificial intelligence (AI) algorithms in medicine have rapidly progressed from theoretical possibilities to exciting real-life applications in everyday use [1]. With the advances in computer processing capacity and the mainstream collection of “big data” sets [2, 3], such applications are finding an ever-widening scope in medical and surgical subspecialties. Specifically, within the field of orthopaedics, AI applications are becoming more and more commonplace [4, 5]. Given that many potential aetiologies ultimately converge leading to consideration for joint replacement surgery, the potential for the application of AI technologies in this area is great. Predicting the differential development of end-stage joint degeneration as a result of common precursor conditions, such as osteoarthritis (OA), systemic inflammatory disorders, trauma, intra-articular infection and dysmorphology is one such opportunity. Some exciting early developments in AI applications already propose accurate prospective determination of the evolution of joint degeneration, even before radiographic or perhaps clinically detectable changes. In this regard, the potential for early targeted intervention and genuinely disease-modifying effect holds much promise. While there appears to be much enthusiasm and many apparent applications of AI within lower limb arthroplasty [6], there are few robust summaries/reviews to provide an evidence-based foundation for the prospective surgeon. Therefore, the aim of this structured review was to examine the contemporary literature regarding AI applications specifically within the domain of total knee arthroplasty (TKA) and to provide the reader with an up-to-date summary of the topic.

## Methods

**Fig. 1:**
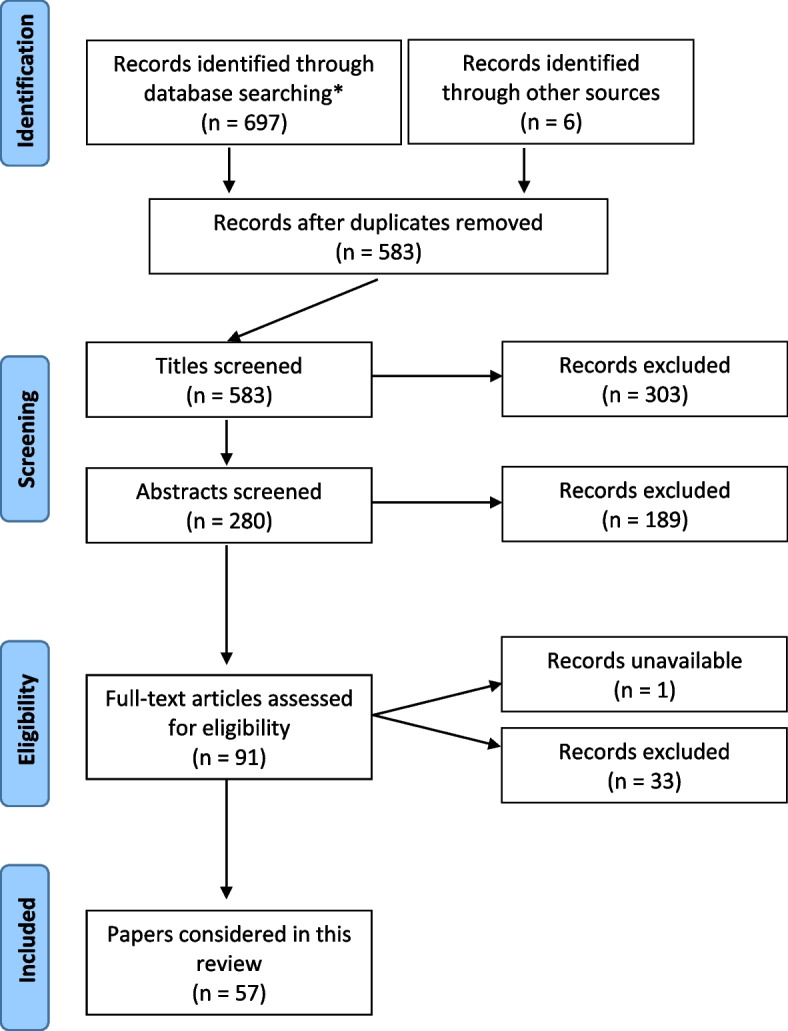
PRISMA search summary

To ensure a relevant, accurate and representative synopsis of the current state-of-understanding of AI applications within TKA surgery, a structured and systematic search and retrieval of publications was performed according to the accepted Preferred Reporting Items for Systematic Reviews and Meta-Analyses (PRISMA) guidelines. The search results are depicted in Fig. 1. Three databases: (I) Cochrane; (II) EMBASE; and (III) Medline were searched from inception until 9 November 2022. Search results were limited in the first instance to articles available in the English language with available abstracts. The following MESH terms were used: “[(knee) AND (arthroplasty OR replacement)] AND [(artificial intelligence OR AI)]”. Titles and abstracts of identified records were screened to exclude obviously irrelevant studies. All articles describing AI during—or in association with knee arthroplasty—were reviewed. No restrictions were placed on age, gender, date, type of study, or length of follow-up. Articles were excluded if they did not specifically discuss the use of AI in relation to knee arthroplasty or if the full text was not available in English. The bibliographies of relevant papers were manually reviewed to identify further studies, with additional data sourced from international joint registries.

Initially, 697 articles were identified during preliminary database searching. After exclusion of duplicates, articles which did not match the search intent (i.e., papers not specifically exploring content related to AI applications associated with TKA) and articles not available in full text form, 91 full text papers were manually reviewed. At the end of the review process, 57 articles were deemed appropriate for inclusion. As a relatively new topic in the field, it was identified that there existed a lack of quantitative research within the domain thus preventing formal “meta-analysis”, per se. With the preserved intent of providing a contemporary synopsis of the topic, a structured review of the identified literature was performed in keeping with meta-synthesis principles.

### Establishing the role of AI in TKA

The concept of AI is widely acknowledged to have been introduced in 1956 [7] defining a computational process in which a machine (i.e., computer) was predicted to perform on some form of iterative process that mimicked elements of human cognition and processing [8], with limited (if any) direct human input [9]. Theoretical concepts soon became practical realities in the years that followed, although computer processing capacity long-remained the rate limiting step to application [7, 8]. With near exponential advances in computer speed and volumetric processing capacity, AI has rapidly become a mainstream utility in many areas of general life, including medicine [8]. Much of the practical value of modern AI has come from areas where a computer can be programmed to examine large volumes of raw or refined data in time frames far beyond exceeding human comprehension [7, 10]. Once programmed instructions (i.e., algorithms) have been established, the computer can be directed to perform highly specific and reproducible tasks [11]. Strengths of such applications to date have included identifying, linking or clustering (categorizing) data variables after sifting through large amounts of information [12–14]—in medicine this has already been shown to be of great value in outcome/risk prediction [15, 16]. With highly-refined coding (i.e., instructions) the algorithm can develop the ability to recognize specific features of the data set and can therefore be considered to be “learning” [17]. Classically, the “accuracy” of the desired key output has been compared to a human “gold standard” (or human-defined expectations), and refinements to the operational algorithm can then be made. However, with increasing computing power and multi-layer processing capacities, the AI system can be “taught” to perform self-evaluation and in turn modify its own internal algorithmic codes [9, 18]. This subset of AI is called deep learning (DL) [12, 17, 19–21]. The system starts with a set of predetermined key outcomes and known, linked, associative variables. It progressively re-refines its ability to associate clusters with each new epoch (i.e., training run), thereby improving accuracy [9]. Modern DL neural networks [22] allow the artificial creation of multi-layered “evolutionary plexuses”, which have been conceptually compared to human neurons [7, 23]. Most modern DL systems consist of some form of artificial neural network (ANN) [14, 17, 24], which in practice represents a series of iterative processing steps between an “input” layer (e.g., where the data under consideration is entered) and a final “output” layer [25]. These complex AI systems are also known as deep convolutional neural networks (CNNs) [26–29].

### Current artificial intelligence applications in TKA

After an extensive review of the available published literature, it appears that there is an apparent enthusiasm for realizing the potential of AI in joint replacement surgery. Most of the applications reported to date that are relevant to TKA appear to exploit the current strength of AI in predicting outcomes or events. With this in mind, four key areas of utility have been recognized: imaging-based/diagnostic applications, medical/adverse event prediction, hospital and administrative considerations, and applications directly related to surgical planning and/or surgical performance. Each of these areas is discussed in sequence below.

#### Imaging

Generically, AI applications into the realm of semi-automated [30] (or fully automated) [31] image feature or pattern recognition represent one of the most successful forays to date [7, 11]. Given the inherent reliance on information garnered from diagnostic imaging studies in many areas of medicine, including surgery, there is a natural opportunity to test and apply AI’s interpretive capabilities in this context. When looking specifically at knee arthroplasty, the majority of reported work to date has been related to either the diagnosis of OA [1, 15, 32, 33] and/or the grading of OA [1] from plain radiographs [34, 35], or the identification of in situ implant components [34, 36–38]. Relatively simple diagnostic analyses based on single AP X-rays have been reported [32, 34], as well as more sophisticated works using customized, multi-planar, imaging protocols [39]. Such applications have largely focused on either diagnostic accuracy or prediction of future OA disease development/evolution [36, 40], often with progression to TKA as a definable endpoint [1, 32, 36, 41]. Capitalizing on the processing power of modern AI algorithms, the addition of key clinical baseline data to the basic digital image provision has been shown to further increase the precision of predictive accuracy [40]. With the touted ability to accurately and reliably detect subtle image features that diagnose early (i.e., “preclinical”) arthritic changes, some authors have suggested an opportunity for broader screening and possibly the initiation of disease-modifying interventions [12]. Such early guided treatment initiation has the potential to alter the underlying degenerative progress [40, 42]. Especially in early stages, the iterative capability of machine learning/computational processing pathways may also allow the identification of non-traditional diagnostic features and allow for permitting earlier diagnosis and/or initiation of treatment [43].

As the evolution of OA considered is a spectrum of disease presentation, a reliable prediction of clinical progression allows for a pre-emptive determination of “when” a TKA may be required [41] and may be of clinical value in informing patient management discussions. Recent work by Houserman et al. [39] using a machine learning (ML) model demonstrated valuable utility in predicting the future need for TKA, UKA or no surgery with multiclass receiver operating characteristic (ROC) curves greater than 0.96. The system provided 94% accuracy in predicting surgery versus no surgery and an 88% overall accuracy in predicting the need for definitive surgical versus non-surgical intervention [39]. Extending this work beyond plain film radiographs, Tolpadi et al. [42] demonstrated that a DL AI model could predict the future need for TKA from conventional MRI imaging with high accuracy (i.e., AUC of 0.94), even in patients without clinical evidence of OA.

The other major area of interest in AI-assisted image analysis has been the area of in situ component identification. There are many clinical circumstances in which details regarding implanted arthroplasty components are not immediately available (or are simply not available at all), but such information is critical for patient management (e.g., planning revision surgery [37, 38]). While reports vary, it has been suggested that in 10% or more of cases, key implants that will be subject to subsequent revision cannot be identified prior to surgery itself [37]. There are many potential benefits to even small improvements in the preoperative capacity to correctly confirm in situ components. First, the correct (or even proprietary) removal/extraction devices can be ordered to facilitate timely and bone-preserving techniques. Second, compatible trials and definitive implants can be ordered, with the possibility of component retention (e.g., during planned DAIR procedures for acute prosthetic infections). Third, the application of accurate AI algorithms in automated procedures is likely to result in significant time savings when compared to traditional human/manual approaches. This translates into both time savings for clinicians [34], but also potentially reduced delays in surgery for such purposes—again with positive resource and cost implications [34], in addition to reduced patient risk and morbidity from otherwise unnecessary delays in surgery. Reducing the incidence of component mismatch also potentially avoids many (often significant) perioperative hurdles.

Modern AI applications have already been reported to be accurate in identifying [15, 33] both the manufacturer and model [34] of in situ TKA components. Using human observers as the comparative standard [36], most AI algorithms report > 90% accuracy [36] and consistently outperform senior orthopaedic specialists in this regard [38]. In an effort to understand the iterative processes underpinning such success, it has been suggested that DL applications likely rely on the identification of specific/unique image features [12, 34], which may already exceed discernible human perceptual abilities. After 1000 training epochs, the DL model constructed by Karnuta et al. [34] showed near-perfect precision in identifying 9 separate implant types with an AUC of 0.99, and resultant accuracy, sensitivity and specificity of 99%, 95% and 99%, respectively [34]. While the limitation of the relatively small numbers of implant types included in most studies reported to date (i.e., usually < 10) is acknowledged [36, 38]—potentially limiting wider external validity [12]—proponents are quick to point out that the iterative capacity of most DL/ML algorithms allows relatively uncomplicated scalable extension to other implants [34].

Other currently novel/less extensively studied imaging-based AI applications in knee arthroplasty include the accurate diagnosis of component loosening from plain film X-rays [33, 36], prediction of postoperative hip-knee-ankle axis (HKAA) [31], determination of mechanical and implant alignment from standing long leg images [10], and the semi-automated 3D reconstruction of sectional CT images to inform subsequent surgical robotic planning [44]. While certainly tantalizing extensions of image-based AI work in knee arthroplasty, further confirmatory evidence is required to support consideration of wider adoption [12].

#### Medical

From a non-surgical, medical perspective the great clinical value of AI applications in knee arthroplasty has largely centred on predicting the outcome of medical events/adverse events. In most instances, such studies to date have focused on a range of recognized perioperative/postoperative adverse events with the purpose of enabling prospective identification of “at risk” individuals and/or to allow the instigation of risk mitigating interventions [45]. Much of the success in this area has been facilitated by retrospective access to “big data” cohort sets (and thus the ability to train robust algorithms), often containing > 10,000 patients [33, 46]. The widespread evolution from paper-based to electronic medical records (EMRs) [47] has often made this a less onerous step. Published reports have demonstrated the value of AI in predicting AKI [48], the risk of perioperative blood transfusion [33, 46, 49], the development of postoperative delirium [47] or ischemic stroke [45], and even the likelihood of persistent or prolonged opioid analgesic requirement [50, 51].

#### Hospital/healthcare

Given the incredibly large volumes of data associated with patient management and episodes-of-care, it is perhaps not surprising that the broad application of AI in arthroplasty at the population level has been heavily investigated in the administrative domain. Leveraging readily available big data sources (e.g., the US National Surgical and Quality Improvement Database, etc. [52, 53]) tens and often hundreds of thousands of patient’s records [26, 52, 53] have been drawn upon to inform AI algorithm training sets. Prediction of inpatient length of stay (LOS) [6, 26, 33, 53, 54], including prediction of same-day discharge likelihood [52], has been extensively studied, largely through associative-clustering. While such work has allowed identification of key considerations associated with postoperative LOS (e.g., patient age > 75, Charlson Comorbidity Index score, BMI, and a number of specific comorbid conditions [54, 55]), in many instances the prospective predictive value of such applications has failed to outperform traditional “human” methods [56].

Beyond the simple determination of likely LOS, other applications have also explored the prediction of discharge destination [3, 22, 33] following both primary and revision surgery [57]. Early work has demonstrated the multifactorial nature of such considerations, highlighting the complex interplay of critical patient factors. Much of this work has been retrospectively validated using institutional or heath network databases and the true prospective predictive value in many instances remains to be independently proven [1]. Similarly, clinical events associated with the prediction of postoperative adverse events [58] and all-cause 90-day unanticipated readmission rates [59] have also been widely considered, although few meaningful studies have been successfully replicated away from the index reporting site. Thus, while such studies provide tantalizing evidence of future value, current generalizability is limited.

Parallel applications have included preoperative prediction of inpatient costs associated with episodes of care [6, 25, 26, 33], which has potential value in resource utilization/allocation [18, 60] and strategic discharge planning [54]. In healthcare systems where reimbursement for clinician and/or institutional services is variable and often tied to a number of episode-of-care related considerations, payment prediction has also become a valuable AI application [25]. Such information can support bundled care payment schemes [26, 61] under the guise of either value-based or patient-specific care pathways [25, 26]. Large-scale work has enabled the identification of value metrics associated with TKA surgery [61] and—through cluster linkage—has allowed accurate determination of graded episode-of-care costs associated with various, common, severe patient comorbidities. Used judiciously, this information can accurately predict the cost of care for specific patient cohorts, recognizing that many patient health and social factors interact to influence this. This information may allow more evidence-based and justifiable allocation of health funding [22].

With the global pressure on elective surgical waiting lists exacerbated by the recent widespread COVID-19 restrictions, novel AI applications have shown promise in the administrative management of the backlog of outpatient and surgical waiting cases [62] and may aid in the nomination of best practice care pathways [62]. Authors of such work advocate the cost, time, and morbidity savings of accurately and timely directing patients to the treatment/healthcare management stream best suited to their clinical needs.

#### Surgery specific applications

Despite the widespread hype, AI applications in TKA that are actually related to the direct delivery or optimization of surgical care remain limited [15]. Again, playing on the recognized strengths of modern ML/DL algorithms in outcome prediction, much of the successful work in this area has been related either to decision-making aids [11, 18, 22, 63] for optimized patient selection [6, 7, 63], patient education [27] and expectation management [30, 64], or to the identification of patients “at risk” of a poor or adverse outcome [15, 50, 59]. For patients in the latter group, such early identification may allow for the implementation of risk-reduction interventions/approaches [51] or support [65]. Index authors suggest that the application of training algorithms in such settings does not increase preoperative consultation time [27] and may be valuable in optimizing postoperative patient satisfaction [1, 27, 33, 66], functional recovery trajectory [11, 17, 27, 30, 65] and formal PROM scores [1, 6, 20, 64].

Surgical and implant planning aids [6, 11, 67] have also been explored with highly demonstrated predictive accuracy [68] and potential time savings [67]. Early work has suggested disproportionate accuracy in prediction of final femoral component sizing although algorithm refinements are likely to show improved utility for tibial component sizing also [67]. Coupled with high-resolution preoperative imaging, AI applications have shown value in identifying anatomical landmarks to guide construct alignment [69], which may benefit the precision obtained intraoperatively.

Individual studies have reported mixed efficacy in predicting the duration of surgery [53], surgical outcomes and complications [1, 60], the risk of infection (especially after multiple surgeries) [70] and early overall revision [21], and the likelihood of catastrophic implant failure [36]. While these are all exciting applications, much work remains to be done to validate such findings validated beyond proof-of-concept reports and to safely apply them in independent, non-designer, settings [68]. An open willingness to make developed DL algorithms available to other interested users will support this, but will ultimately be weighed up against competing commercial and intellectual property considerations.

## Discussion

The world around us continues to change at an incredible pace, and technological advancement continues to be a part of this. Not just in the realm of highly-specialized robotics and space ship applications, the creeping invasion of computers and AI into our everyday lives is widespread and long-standing. Many are surprised to be reminded that the AI-driven “Google search” function came into mainstream use 25 years ago (1998) [18]. Patients are living longer, with ever-increasing expectation for sustained activity and quality of life [71–73]. Similarly, AI has found its way into medical and surgical fields, as it has into lower limb and indeed knee arthroplasty [1, 2, 16]. Having completed a comprehensive review of the published literature, it is clear that much of the work available for review represents either proof-of-concept studies and/or the enthusiastic early results reported by algorithm creation sites. There exists scarcely little evidence of independent replication or validation of results in non-designed settings—as in any other clinical domain, this is a concern and greatly limits the evidence base that would otherwise be required to support wider adoption. While sometimes applications have sometimes been tested against accepted human “gold standards”, in many cases no clear (or fair) head-to-head comparison has been made. As such a number of studies are best classified as “demonstration of concept” rather than even “proof of concept”, as is often touted [5, 20]. No high-level research evidence (i.e., no Level I or II studies) was identified in this emerging topic area, with limited Level III work available. The majority of the identified papers could best be considered as Level VI evidence (i.e., evidence from single descriptive or qualitative studies). As with any emerging/novel topic area, this lack of quantitative evidence will undoubtedly redress with time as further validation of early works and wider application occurs. While there has been much enthusiasm in the reported literature to date attempting to demonstrate value-added AI applications, it is important to recognize that not all results have been good [6, 21], and that such algorithms have often failed to outperform (or even match) existing “human-driven” approaches [17, 44, 74, 75].

Most of the published work can be grouped into one of four common areas: imaging-based/diagnostic applications, medical/adverse event prediction, hospital and administrative considerations, and surgical planning and/or operative performance. There appears to be little overlap between the domains, and an apparent lack of cross-collaboration between individual research efforts was noted. Most groups appeared to be working in silos—seemingly “starting from scratch” rather than showing evidence of “building on the work of others”.

This review highlights many common shortcomings: first, there are many gaps in the current knowledge base regarding AI applications in TKA [1] that remain to be filled. Reported cohort sizes are often small [27, 32] and almost uniformly retrospective in nature, limiting generalizability/validity. Many reported studies are at high risk of confounding bias [36]. There is an urgent need to improve reporting standards [4] and data collection quality [5, 74] so that higher quality outputs can be more easily and consistently achieved. Larger (ideally prospective) studies are needed [48] with larger training datasets [1] to allow optimized algorithm refinement before extension to “real life” applications.

While most authors agree that AI/ML applications have the potential to rapidly improve the science, economics and delivery of lower limb arthroplasty [2], extensive further study is still needed [1, 36, 66, 76]. AI has already shown much promise in TKA surgery [30] and has the potential to improve outcomes [77] and may help to identify/develop novel solutions to long-standing problems [16]. There is hope that AI may play a valuable role in reducing the recognized element of patient dissatisfaction after surgery [30, 78], with most authoritative sources still suggesting that approximately 15%–20% of patients report being “unhappy” with their TKA [65, 76, 78]. This is likely to be achieved through a combination of optimized patient selection and better executed (i.e., AI-informed) surgical care.

While AI will certainly expand the boundaries of orthopaedic surgery [2], it is unlikely to replace human experience and traditional methods in the near future [7]. While the value is clear for simple tasks (particularly volumetric processing applications), the ability of ML systems to accurately predict complex interactive outcomes remains imprecise [16, 33]. To facilitate the broader translation of AI models to real-world conditions [36], there is continued and increased collaboration between surgeons and scientists [16], while maintaining the common goals of benefit to the end user (i.e., the patient). Undoubtedly, AI applications in TKA represent a thriving and exciting area of contemporary medicine [71, 79], but there remains a need and responsibility to ensure that the scientific evidence base [36] that underpins the growth and adoption of such work is not overtaken by overly enthusiastic hype.

## Conclusions

Despite widespread enthusiasm for the use of AI applications in knee arthroplasty, the evidence base to support such endeavours remains limited. Much of the quality work to date has focused on risk, cost and outcome prediction—rather than actual surgical applications, per se. As this review highlights, many of the findings reporting the use of AI lack independent external validation in non-designer sites, and the transparent generalizability to non-specialist centres is largely absent. Much of the published work suffers from confounding methodological limitations and biases, and inconsistencies in the purity of critical data sources are also concerning.

In a practical sense, in areas requiring the processing of large amounts of collected data, with relatively simple desired outputs, AI has often performed well and is likely to offer great time efficiency and consistency. However, for more complex tasks, AI has often failed to match the accuracy and reliability of traditional human-driven standards. As has been seen in other areas of medical (and non-medical) life, it is likely that AI will continue to be refined and advanced to find consistent value in many aspects of TKA. However, much high-quality work remains to be done to provide a robust evidence base to support consideration for more mainstream use and to ensure that the science behind such technologies keeps up with the hype.

## Data Availability

Not applicable.

## Abbreviations

3D: Three dimensional
AI: Artificial intelligence
AKI: Acute kidney injury
ANN: Artificial neural network
BMI: Body mass index
CNN: Convolutional neural networks
COVID-19: Coronavirus disease
CT: Computed tomography
DAIR: Debridement, antibiotics and implant retention
DL: Deep learning
EMR: Electronic medical record
LOS: Length of stay
ML: Machine learning
MRI: Magnetic resonance imaging
OA: Osteoarthritis
PRISMA: Preferred Reporting Items for Systematic Reviews and Meta-Analyses
PROM: Patient-reported outcome measure
ROC: Receiver operating characteristic
TKA: Total knee arthroplasty
UKA: Unicompartmental knee replacement
US: United States
